# Persistent Genital Arousal Disorder: Confluent Patient History of Agitated Depression, Paroxetine Cessation, and a Tarlov Cyst

**DOI:** 10.1155/2014/529052

**Published:** 2014-11-27

**Authors:** Simone Eibye, Hans Mørch Jensen

**Affiliations:** Psychiatric Centre of Copenhagen, Bispebjerg Bakke 23, 2400 Copenhagen NV, Denmark

## Abstract

We report a case of a woman suffering from persistent genital arousal disorder (PGAD) after paroxetine cessation. She was admitted to a psychiatric department and diagnosed with agitated depression. Physical investigation showed no gynaecological or neurological explanation; however, a pelvic MRI scan revealed a Tarlov cyst. Size and placement of the cyst could not explain the patient's symptoms; thus neurosurgical approach would not be helpful. Her depression was treated with antidepressant with little effect. Electroconvulsive therapy improved the patient's symptoms though they did not fully resolve. More awareness of PGAD and thorough interdisciplinary conferences are necessary to insure an unequivocal treatment strategy.

## 1. Background

Persistent genital arousal disorder (PGAD) is a rare disease. Only a few case reports have been published [1–4] and no prevalence has been reported yet. It is often women who suffer from the symptoms of PGAD; however, the diagnosis is not gender-specific. The symptoms of PGAD are as follows [5]:

1. symptoms characteristic of sexual arousal (genital fullness/swelling and sensitivity with or without nipple fullness/swelling) which persist for an extended period of time (hours to days) and do not subside completely on their own,
2. symptoms of physiological arousal which do not resolve with ordinary orgasmic experience and may require multiple orgasms over hours or days to remit,
3. symptoms of arousal which are usually experienced as unrelated to any subjective sense of sexual excitement or desire,
4. the persistent genital arousal which may be triggered not only by a sexual activity but seemingly also by nonsexual stimuli or by no apparent stimulus at all,
5. symptoms which are experienced as unbidden, intrusive, and unwanted,
6. the symptoms which cause the patient at least a moderate degree of distress.

PGAD was previously called persistent sexual arousal syndrome (PSAS) and was first described by psychiatrists Leiblum and Nathan in 2001 based on internet surveys [6]. It was subsequently renamed as PGAD by Goldmeier et al. in 2009 [5]. The origin of PGAD is yet unknown; however, it has been described after cessation of selective serotonin reuptake inhibitor (SSRI) medication and selective serotonin-noradrenalin reuptake inhibitor (SNRI) medication [3–5, 7]. Also, it has been described as correlated to anxiety [8, 9], somatizing stress [9], increased soy intake in diet [1], and small fiber neuropathy [7] and a few cases have reported Tarlov cyst as the aetiology of PGAD symptoms [10]. Waldinger and Schweitzer [7] suggested to rename the disorder: Restless Genital Syndrome because of its overlapping symptoms with restless legs syndrome (RLS) [11].

## 2. Presentation of the Case

In 2008, a 31-year-old woman was referred to the out-patient psychiatric department with anxiety, depression, and somatization by a psychiatrist in private practice. She had a history of anxiety, family depression, violence, and inappropriate touching in the childhood and had earlier demonstrated a potential for benzodiazepine abuse. She joined group therapy but quit due to low compliance. Afterwards she received individual therapy sessions with a psychologist and was also treated by a psychiatrist, though she did not improve significantly. She described her relationship with her mother as symbiotic and dependent. Four years before the PGAD symptoms arrived, her mother died of colon cancer and the patient participated in grief group therapy. In 2010, the patient was diagnosed with a dependent personality disorder. She was prescribed different medications for depression and anxiety (paroxetine, sertraline, citalopram, fluoxetine, duloxetine, venlafaxine, mianserin, amitriptyline, nortriptyline, pregabalin, gabapentin, chlorprothixene, quetiapine, and benzodiazepine); however, due to lack of compliance, the only antidepressant taken as prescribed was paroxetine. She described increased sexual symptoms from July 2011. In November 2011, the symptoms increased and she was seen by a psychiatrist at the Department of Sexological Research at Rigshospitalet, Copenhagen. She presented with the six symptoms of PGAD and was diagnosed with PGAD. Though the patient had been treated with several psychotropics with different mode of action, she persistently claimed that the first symptoms of PGAD were timely related to cessation of paroxetine. Afterwards, she restarted paroxetine but did not achieve complete remission. The PGAD symptoms increased even further when agomelatine was administered. Since the patient tried several other pharmaceuticals, a causal relationship with one or several other psychotropics cannot be ruled out. Anxiety caused her genital symptoms to increase and she was introduced to mindfulness therapy [12]. Psychological treatment was intensified, yet her symptoms of PGAD increased.

## 3. Investigations

She was admitted to an in-patient psychiatric department in June 2013 and an extensive somatic examination was initiated. She had already been examined by a gynecologist and a gynecological examination including ultrasound revealed no pathology, including clitoral priapism. Additionally, a pudendal block was performed without relief of symptoms. Investigation for RLS was inconclusive. The thyroid and sexual hormones were explored by mid cycle blood samples: FSH, LH, progesterone, estradiol, prolactin, testosterone, TSH, T3, and T4 were all normal. The patient had no excessive soy intake in her diet. The patient was also seen by a neurologist who found her neurologically intact. Local anesthesia was attempted without improvement. As her symptoms became increasingly unbearable, she was referred to physiotherapy but the exercises only improved the symptoms briefly. Anxiolytic and sleep medications (benzodiazepine, triazolam, melatonin, and zopiclone) were administered with effect but, with time, increasing doses were needed to provide the patient relief. She was consulted and examined by different psychiatrists and a conference discussion relieved that several doctors had observed somatization. She was diagnosed with agitated depression and treated with mirtazapine. Her symptoms did not improve remarkably on this treatment and she was constantly tormented by the symptoms. Tramadol was attempted when symptoms were unbearable. A review of the literature suggested offering the patient electroconvulsive therapy and, after a couple of treatments, the symptoms of PGAD improved and periodically resolved completely. However, after five treatments, she refused further therapy and the symptoms slowly relapsed. A magnetic resonance imaging (MRI) revealed a Tarlov cyst in relation to S2 and S3. The cyst was 1∗1∗1 cm with homogeneous fluid; also a discrete spina bifida was visualized in the lumbar area of the spine. The MRI was reviewed by the neurologists and neurosurgeons who found the symptoms unlikely to be caused by the cyst or spina bifida. The patient was discharged from the psychiatric department at her own request but she kept attending out-patient individual therapy sessions. Four months later the patient revealed an increasingly daily use of morphine, tramadol, and benzodiazepines prescribed by different doctors in neurology, psychiatry, gynecology, anesthesiology, and general practice.

## 4. Differential Diagnoses

Persistent genital arousal syndrome is a diagnosis of exclusion and, therefore, thorough physical investigations are crucial. Pelvic MRI was performed to rule out pelvic masses; however, MRI of the brain and electroencephalogram (EEG) were not offered. Hormonal status ruled out early menopause as well as thyroid disorder. Clitoral priapism is a rare diagnosis, which causes severe pain and swelling of clitoris [2]. A vaginal inspection was performed when our patient experienced unbearable symptoms and clitoral priapism was ruled out. Investigations for RLS were inconclusive; however, the patient was treated with oxazepam and tramadol with some effect. These medications have been described as effective against RLS as well as for PGAD [7]. Notable was the fact that the patient had also been treated with agomelatine for which RLS is registered as a rare side effect (0.1–1%) [13]. Waldinger and Schweitzer suggest PGAD as part of a cluster syndrome including overactive bladder but the described case did not suffer from overactive bladder [7]. As with a number of other diagnoses of exclusion, the aetiology of the patient's symptoms is inconclusive. The symptoms presented after paroxetine cessation and though a Tarlov cyst was located, none of these aspects offer sufficient evidence of causality.

## 5. Treatment

The patient's symptoms resolved briefly after physiotherapy, anxiolytic drugs, morphine administration, mindfullness, and electroconvulsive therapy. Several medications have been administered without effect; thus, besides paroxetine and mirtazapine, the patient was not compliant. Since her condition did not improve at the open psychiatric ward, she has been offered and is considering admittance to the intensive psychiatric ward for rightfully medical administration to treat her depression state. She still attends individual therapy sessions; however, her symptoms are progressing and her suicidal thoughts are slowly intensifying. Interdisciplinary conference is crucial to ensure an unequivocal treatment strategy for patients with low compliance and potential abuse. Follow-up was ended early March 2014.

## 6. Discussion

We report a case of PGAD after paroxetine cessation. The patient suffered from agitated depression and additionally a Tarlov cyst. Symptoms improved during a brief series of electroconvulsive therapy; however, they did not fully resolve. Yero et al. reported two cases of PGAD where the symptoms resolved after electroconvulsive therapy, though these women also suffered from bipolar disorder [4]. Korda et al. described a case of PGAD after abrupt paroxetine cessation which benefitted from electroconvulsive therapy; however, remission was only short-lived before relapse [3]. Excessive soy intake has been associated with PGAD with full remission after abstaining from soy intake but our patient did not report excessive intake therefore; her condition cannot be explained by the association [1].

Komisaruk and Lee suggested to rule out Tarlov cyst for patients suffering from PGAD [10]. We performed an MRI which revealed a Tarlov cyst. The prevalence of Tarlov cysts in the background population has been reported as 1.5% [14], whereas a recent internet-based study reported the prevalence of Tarlov cysts for women with PGAD as 66.7% [10]. The women were invited to submit MRIs of their sacral region through a PGAD support group. It has been reported that the majority of Tarlov cysts are incidental findings [14, 15]. A clinical study of 138 women and 19 men with Tarlov cysts verified on MRI [16] tested the patients for bladder symptoms and performed a complete electroneurography as well as depression rating. Female participants were also interviewed about gynaecological symptoms. The patients suffered from sexual distress in 28.2% (female) and 36.8% (male) of the cases. The Hamilton Rating Scale for Depression (scale type not specified) revealed a nongender-specific mild depression. Notable is the fact that the study did not use a matched reference group. In 2007, Leiblum et al. investigated sexual symptoms in 388 women from an online anonymous internet survey [9]. Of these women, 206 suffered from PGAD while the remaining 182 women had sexual symptoms; however, they did not meet the criteria of PGAD. They noticed that more women with PGAD suffered from somatising stress which also was noticed for our patient.

Depression and anxiety have been suggested to maintain PGAD symptoms [17, 18]. Our patient both suffered from depression and anxiety. She also had a history of inappropriate touching in the childhood and also experienced low sexual interest as adult. A recent study performed a web survey and included 43 women with self-described symptoms of PGAD and 42 controls [19]. The patients' questionnaires reported a less liberal sexual script for women with self-reported PGAD. Notable is the fact that the women were never seen by a medical doctor; therefore, differential diagnoses were never excluded.

Our patient suffered from sexual symptoms after paroxetine cessation. Psychiatric investigation revealed an agitated depression and comorbid anxiety for which mirtazapine and antianxiety drugs (i.e., benzodiazepines) were prescribed with some effect. A Tarlov cyst was also located; however, a neurosurgical evaluation found that size and placement could not explain the patient's symptoms. The patient benefitted from electroconvulsive therapy but none of the above mentioned aspects offer sufficient evidence of causality.

## 7. Bullet Points

1. Persistent genital arousal syndrome is a diagnosis of exclusion; therefore, thorough physical investigations are crucial.
2. More awareness to the diagnosis will ensure faster treatment and reduce patients' agony.
3. To ensure an unequivocal treatment strategy, interdisciplinary conference and a case manager should be suggested.

## Supplementary Material

Timeframe of our patient's history' Supplementary text could be: 'The patient had a thorough physical and psychiatric evaluation during the diagnostic process'.

## Abbreviations

PGAD: Persistent genital arousal disorder
MRI: Magnetic resonance imaging
PSAS: Persistent sexual arousal syndrome
SSRI: Selective serotonin reuptake inhibitor
SNRI: Selective serotonin-noradrenalin reuptake inhibitor
RLS: Restless legs syndrome
FSH: Follicle-stimulating hormone
LH: Luteinizing hormone
TSH: Thyroid stimulating hormone
T3: Triiodothyronine
T4: Thyroxine
EEG: Electroencephalography.
