# Supplementary material for: Persistent Genital Arousal Disorder: Confluent Patient History of Agitated Depression, Paroxetine Cessation, and a Tarlov Cyst
Source: Case Rep Psychiatry. 2014 Nov 27;2014:529052. doi: 10.1155/2014/529052 (PMC4265540; doi:10.1155/2014/529052)
Supplement: Supplementary file 1 — Timeframe of our patient's history' Supplementary text could be: 'The patient had a thorough physical and psychiatric evaluation during the diagnostic process'. [file 529052.f1.pptx]

## Slide 1
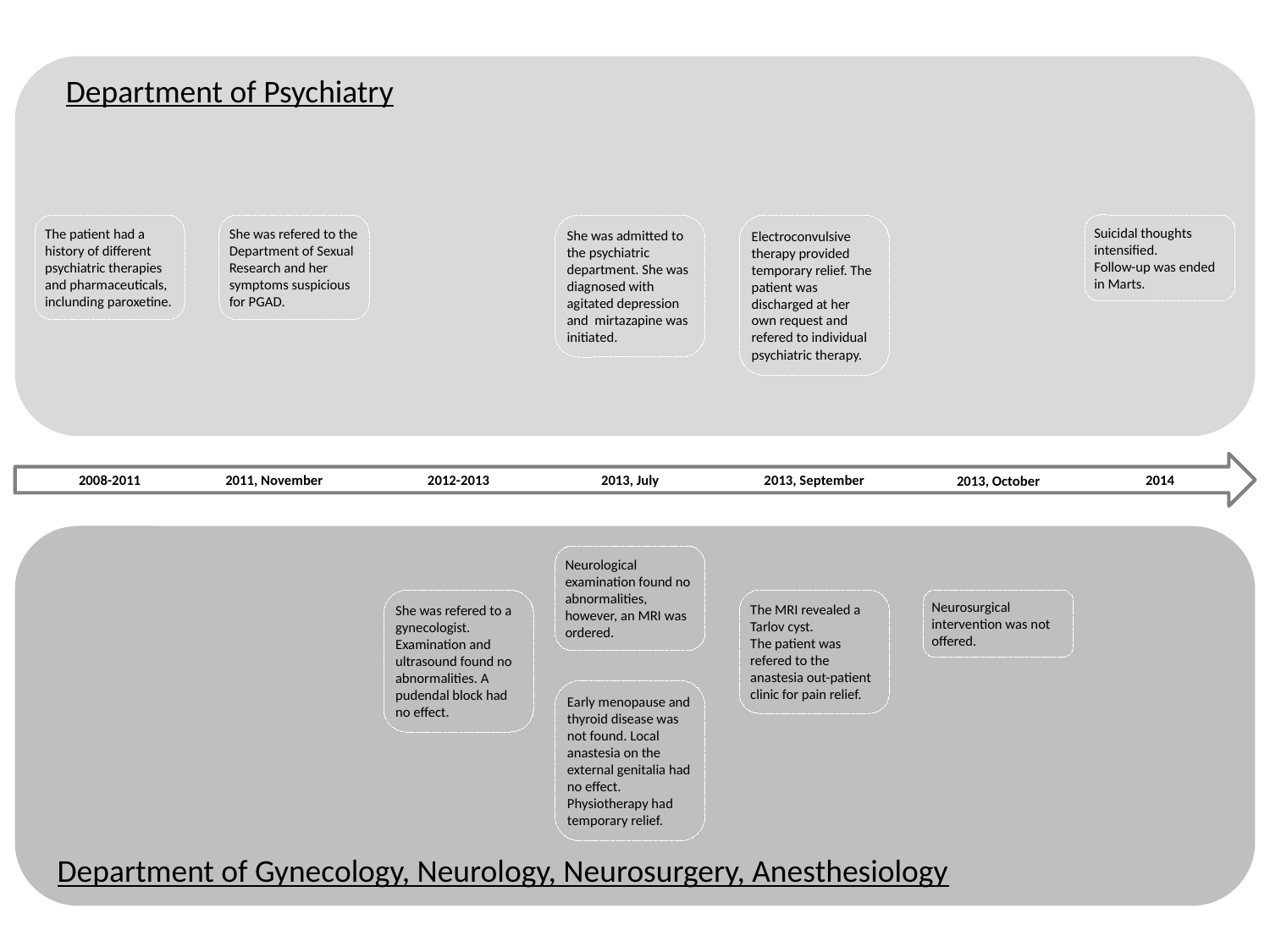

Department of Psychiatry
The patient had a history of different psychiatric therapies and pharmaceuticals, inclunding paroxetine.
She was refered to the Department of Sexual Research and her symptoms suspicious for PGAD.
She was admitted to the psychiatric department. She was diagnosed with agitated depression and mirtazapine was initiated.
Electroconvulsive therapy provided temporary relief. The patient was discharged at her own request and refered to individual psychiatric therapy.
Suicidal thoughts intensified.
Follow-up was ended in Marts.
2008-2011
2011, November
2012-2013
2013, July
2013, September
2014
2013, October
Neurological examination found no abnormalities, however, an MRI was ordered.
She was refered to a gynecologist. Examination and ultrasound found no abnormalities. A pudendal block had no effect.
The MRI revealed a Tarlov cyst.
The patient was refered to the anastesia out-patient clinic for pain relief.
Neurosurgical intervention was not offered.
Early menopause and thyroid disease was not found. Local anastesia on the external genitalia had no effect. Physiotherapy had temporary relief.
Department of Gynecology, Neurology, Neurosurgery, Anesthesiology
